# Effects of Eccentric Single-Leg Decline Squat Exercise on the Morphological and Structural Properties of the Vastus Lateralis and Patellar Tendon

**DOI:** 10.3390/ijerph17249410

**Published:** 2020-12-15

**Authors:** Pablo Abián, Fernando Martínez, Fernando Jiménez, Javier Abián-Vicén

**Affiliations:** 1 Faculty of Humanities and Social Sciences, Comillas Pontifical University, 28049 Madrid, Spain; pabloo9@hotmail.com; 2 Performance and Sport Rehabilitation Laboratory, Faculty of Sport Sciences, University of Castilla-La Mancha, 45071 Toledo, Spain; fermasa83@gmail.com (F.M.); josefernando.jimenez@uclm.es (F.J.)

**Keywords:** eccentric exercise, single-leg decline squat, patellar tendon, vastus lateralis

## Abstract

The purpose was to examine the effect of 6-week eccentric single-leg decline squat (SLDSe) training with two technical execution times (3 s or 6 s) on changes related to the structural properties of the vastus lateralis (VL) and patellar tendon (PT). Thirty-six physical active volunteers were randomly divided into three groups: control group (CG, *n* = 13, age = 20.8 ± 1.9 years, no intervention program), experimental group 1 (EG1, *n* = 11, age = 21.6 ± 2.5 years, execution time = 6 s) and experimental group 2 (EG2, *n* = 12, 21.1 ± 1.2 years, execution time = 3 s). Participants completed a 6-week SLDSe training program (80% of 1-RM) three days a week. The structural characteristics of the VL and the PT were measured with ultrasonography before and after 6-week SLDSe training and after 6 weeks of de-training. Our results indicate that EG1 increased ≈21.8% the thickness of the PT and EG2 increased ≈15.7% the thickness of the VL after the 6-week intervention program. EG1 and EG2 showed greater values (*p* < 0.05) of lean mass and lower values (*p* < 0.05) of fat percentage on the thigh after the intervention program. In conclusion, the SLDSe training carried out with the execution time of 6 s had greater effects on the structural and elastic properties of the PT, and the exercise with the execution time of 3 s caused greater structural adaptations in the VL musculature.

## 1. Introduction

In recent years, the analysis of the use of eccentric exercises as a prevention and treatment modality for the recovery of injuries, mainly muscle and tendon injuries, has increased in the scientific literature [1,2]. Furthermore, eccentric exercises have been introduced into sports training programs due to the physiological characteristics provided by eccentric contractions [3]. Eccentric training can lead to greater strength gains because it implies a lower energy cost to develop a certain load [4]. In addition, there are several mechanisms by which eccentric exercises can lead to better results than concentric training in hypertrophy [5,6].

The effects of eccentric exercises on the neuromuscular system have been evaluated in different studies [7,8]. Knee extensors are the most frequently studied muscle group due to their clinical importance in human locomotion [9], showing a significant increase in muscle strength and lean mass [10,11]. Long-term eccentric exercise programs are characterized by giving rise to a series of functional adaptations that appear in the muscle. Taken together, these adaptations can have important applications for injured people or for those athletes who want to improve their performance. Since muscle is capable of generating more strength in the eccentric phase of contraction than in the concentric phase [12], one of the goals of eccentric training may be to improve muscle strength. On the other hand, the influence of eccentric exercises on healthy or pathological tendons has been less studied than on muscle tissue.

Several authors [13,14] demonstrated that a training program of 6–12 weeks of duration, performing 2–3 sessions a week of eccentric exercises can provoke enough stimulation to improve muscle function in different types of populations. Previous studies assessed the magnitude of muscle strength retention up to 6 weeks of detraining in subjects with moderate physical activity [15,16], and for these studies, the muscular strength returns to control levels over several weeks of detraining via a reversal of the neuromuscular and hormonal adaptations that occurred during the training phase [17]. However, we have not found any study comparing the effects of single-leg decline squat (SLDS) exercise performed in the eccentric phase (SLDSe) on the knee extensor apparatus and the consequences of 6 weeks of detraining. Therefore, we decided to investigate the effects of the SLDSe with different technical execution times (3 s and 6 s) on the morphological and structural properties of the vastus lateralis (VL) and the patellar tendon (PT), which are very important in the knee extensor apparatus. The purposes of this study were (1) to establish and compare which eccentric technical execution time in the SLDSe (3 s or 6 s) causes greater adaptations in the morphological and structural properties of the VL muscle and PT and on the composition of the thigh; and (2) to assess the effect of six weeks of detraining in the variables analyzed of the VL and PT.

## 2. Materials and Methods

### 2.1. Subjects and Inclusion Procedure

Fifty subjects belonging of the University of Castilla-La Mancha were included in the final selection to participate in this study. All of them were tested according to the inclusion and exclusion criteria designed for this investigation. The inclusion criteria were (1) to perform moderate physical activity (3–6 h per week), (2) to be men between 18 and 35 years old, because load response may deteriorate with age (Reeves et al. 2004) and (3) to score > 90 points in the Victorian Institute of Sport Assessment—patellar tendon questionnaire (VISA-P) in its adapted Spanish version (VISA-P-Sp) [18] to rule out symptoms of patellar tendinopathy. Participants were excluded from the study who (1) had had some type of injury to both lower limbs during the 8 weeks prior to start of the study; (2) had performed strength training in the lower body during the 8 weeks prior to start of the study; (3) practiced sports where jumping was a specific action (volleyball, basketball, high jump) more than two hours a week or competitively; (4) consumed supplements aimed at increasing muscle mass and improving strength; and (5) consumed more than 60 mg of caffeine per day (≈1 cup of coffee).

**Figure 1:**
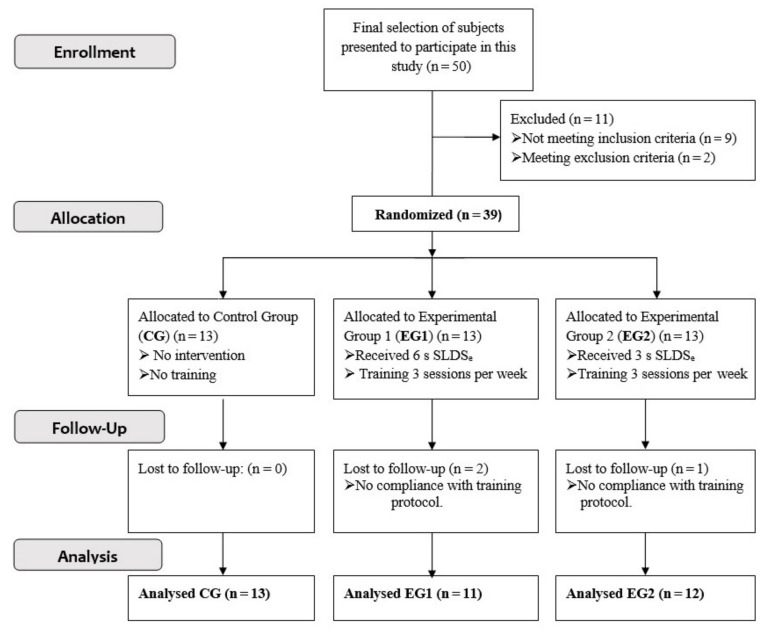
Flow diagram. CG = control group; EG1 = experimental group 1; EG2 = experimental group 2.

The final sample of the study was composed of 39 participants. All of them participated voluntarily and signed the informed consent before the start of the investigation. The participants were randomly divided into 3 groups: control group (CG) made up of 13 subjects, who did not carry out the intervention program; experimental group 1 (EG1) formed by 13 subjects (2 were lost during the follow-up of the study), who carried out the intervention program for 6 weeks, performing the eccentric repetition of the SLDSe during 6 s; and experimental group 2 (EG2) formed by 13 subjects (1 was lost during the follow-up of the study), who carried out the same eccentric training program as EG1 but eccentric repetition of the SLDSe during 3 s (Figure 1). The sample size was calculated beforehand based on previous research [19], which measured the influence of a 6-week eccentric training program on VL thickness. The minimal number of subjects required to attain a power of 0.9 and a bilateral alpha level of 0.05 was calculated to be 8 participants per group. The descriptive characteristics of the subjects can be seen in Table 1.

This study was approved by the Department of Physical Activity and Sport Sciences of the University of Castilla-La Mancha and by the Clinical Research Ethics Committee of the health area of Toledo (number 62, dated 10 June 2015), according to the principles of the latest version of the Declaration of Helsinki.

### 2.2. Design and Procedure

**Figure 2:**
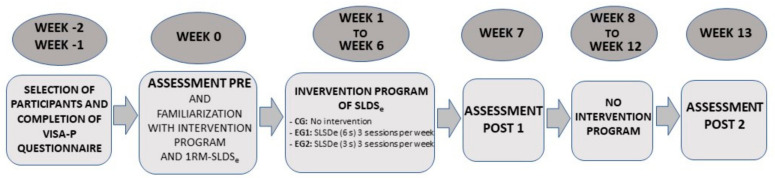
Design of the investigation.

Three assessments were carried out with each of the groups studied (CG, EG1, and EG2) in this investigation. The first assessment was carried out before the intervention program began (PRE) (week 0), the second assessment was carried out at the end of the intervention program (POST 1) (week 7), and the third and last assessment was carried out 6 weeks after the intervention ended to evaluate the residual effects after a 6-week non-training process (POST 2). In the non-training process, participants were instructed to continue their normal lives without participating in any additional training programs (week 13) (Figure 2).

The first session was for data collection (assessment PRE) of the studied variables of body composition by densitometry and of the morphological and elastic variables by ultrasound and sonoelastography so that the sessions of familiarization did not influence these variables. The familiarization sessions and before assessment PRE were to eliminate any learning effects and to inform subjects about the eccentric training program (SLDSe), and 2 days later, the calculation of 1-RM of eccentric exercise (SLDSe) for EG1 and for EG2 in the dominant limb (leg with which the subjects kicked a ball) was carried out in order to determine the training load. The two experimental groups (EG1 and EG2) performed each of the eccentric repetitions in a time of 6 s (EG1) or in a time of 3 s (EG2), resting 6 s between each repetition and reaching up to 90° of knee flexion, which was evaluated with a manual goniometer placed on the joint that followed the bony lines of the femur and fibula. To do this, the participants performed a series of 5 eccentric repetitions of increasing intensity, starting with the weight recorded on the last day of the familiarization pre-testing. With a 2 min rest between each set, the load was increased to establish the 5-RM. In the event that the subject did not maintain the execution speed or did not reach 90° of knee flexion, the repetition was considered null. In the event that there was muscle failure in which the participants could not perform the repetition due to fatigue or inability to tolerate the load, the weight lifted in the last series of 5-RM performed was recorded. If after performing 5 sets it was not possible to obtain the 5-RM, the test was canceled and had to be repeated after 48 h. In this way, we avoided the effect of fatigue on the test result. When the participants reached 90° knee flexion during the exercise, two researchers were placed on each side of the multipower bar, lifted the weight toward the initial position, and the participant returned to the beginning of the exercise using bipodal support. In this way, we reduced the influence of the concentric phase force of the anterior thigh muscles. The time of execution of the eccentric action and the rest time between each repetition were controlled by a metronome (www.webmetronome.net) that emitted acoustic signals indicating the start and end of each contraction. The attainment of 90° of knee flexion was controlled by the principal investigator, placing the goniometer on the knee and determining the end of the repetition.

The 5-RM eccentric test of SLDSe (the same eccentric exercise to be performed in the intervention program) was carried out on a multipower machine (Technogym, Gambettola, Italy). The weight of the 5-RM for each subject was noted, and the calculation of the 1-RM was performed indirectly with the following formula $predicted\;1-RM=\frac{Weight\;Lifted}{1.0278\;-\;\left(0.0278\;\times \;5\right)}$ [20]. In the 6 weeks of the intervention program, the 5-RM eccentric test of SLDSe was repeated by EG1 and EG2 every 2 weeks with the specific training execution time (3 or 6 s) in the dominant limb in order to update the training loads.

The intervention program for both groups (EG1 and EG2) lasted 6 weeks. Every week, 3 training sessions were carried out separated by at least 48 h. All sessions were carried out on the same multipower machine and were supervised by the main researcher and two collaborators. In each of the sessions, a 10 min warm-up was performed on a cycle ergometer at an intensity of 100 W and at a cadence of 80–90 rpm (Wattbike cycle-ergometer, Wattbike Pro, Nottingham, UK). After the warm-up, and for both experimental groups, 3 series of 8 repetitions of the SLDSe eccentric exercise described by Purdam, Jonsson [21] were executed. The rest between repetitions was 6 s and between series was 2 min, and the intensity was 80% of the 1-RM, which was calculated as mentioned above for each group in their specific working technical execution time.

### 2.3. Outcome Measures

An experienced sports traumatologist with extensive musculoskeletal ultrasound training (FJ) carried out all ultrasound examinations on the dominant leg. Morphological examinations of the VL muscle and PT were performed with a Logiq^®^ S8 ultrasound (GE Healthcare, Milwaukee, WI, USA) with a 10 MHz linear probe (ML6-15-D; General Electric Healthcare system). In addition, the elastography index (EI) of the PT was recorded with a probe to measure sonoelastography connected to the same ultrasound machine with which measurements of morphological characteristics were performed (Logiq^®^ S8). Muscle architecture measurements of the VL were performed at two points: 50% of the total thigh length [22] and 4 cm from the distal myotendinous junction. The ultrasound probe was aligned with the fascicle direction to measure thickness and pennation angle of the VL. The assessment of muscle thickness, pennation angle, and fascicle length were performed as described in previous studies [23,24,25].

The PT was scanned in the sagittal and axial planes, taking care to avoid anisotropy. The thickness and the sonoelastography of the tendon were measured at 50% of the tendon length (distance between the lower pole of the patella to the deep distal insertion in the tibia). Sonoelastography was performed by applying light repetitive compression with the hand-held transducer. The elastogram appeared within a rectangular region of interest (ROI) as a translucent color-coded real-time image superimposed on the B-mode image [26]. The color code indicated the strain of the tissues within the ROI, where red corresponded to soft elasticity, green and yellow indicated medium elasticity, and blue indicated hard elasticity. The B-mode image and elastogram were displayed side-by-side on the screen and the graph that appears on the screen standardized the amount and uniformity of compression. The best cine image derived from at least three compression–relaxation cycles was used for the assessment of the EI [26]. A higher value of EI Is related to a higher stiffness level.

The measurements were always taken with the subjects lying down. The VL and PT were scanned with the subject in a supine position and the knee flexed at 15° (0° corresponding to full extension of the knee) with a pillow underneath [27]. All images were analyzed with Image analysis software (Image J, 1.47v, National Institute of Health, MD, USA) to measure fascicle length, pennation angle, muscle thickness, and tendon thickness. The reliability of this ultrasound technique for measurements of human muscle architecture has been previously studied with intraclass correlation coefficients that ranged from 0.92 to 0.99 [28].

Body composition (fat mass, lean mass on the thigh, and fat mass on the thigh) was assessed by dual emission X-ray absorptiometry (DXA, GE Healthcare, Lunar, Diegem, Belgium) with participants in a supine position as previously stated [29]. Thigh-specific analyses were performed as described by Alegre et al. [30], who found a high reliability of this technique with a coefficient of variation that ranged from 0.4% to 3.9%.

### 2.4. Statistical Analysis

The statistical analysis was performed with IBM SPSS Statistics 23.0 (SPSS, Chicago, IL, USA). All data were expressed as mean ± standard deviation. The data were tested for normality with the Shapiro–Wilk test. Since the assumption of normality (all variables *p* > 0.05) was verified, a two-way (3 × 3) repeated measures ANOVA was used to determine the main effects of the two training interventions upon measures of PT variables, VL variables, and the composition of the thigh parameters. One factor was the group (CG, EG1, and EG2) and the other was the timeline (PRE-, POST-1 and POST-2). Effect size statistics were used to quantify the magnitude of the difference in pairwise comparisons, according to the formula proposed by Cohen [31]. The magnitude of the effect size was interpreted using the scale of Cohen [31]: an effect size lower than 0.2 was considered as small, an effect size around 0.5 was considered as medium, and an effect size over 0.8 was considered as large. A probability level of *p* < 0.05 was defined as statistically significant.

## 3. Results

### 3.1. Morphological and Elastic Properties of PT

**Figure 3:**
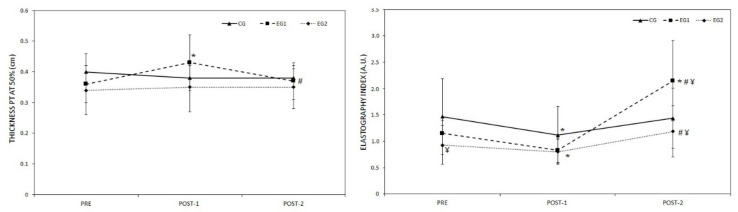
Morphological and elastic properties of patellar tendon (PT) in response to six weeks of eccentric single leg decline squat exercise training (Post-1) with two technical execution times (EG1 = 6 s and EG2 = 3 s) and 6-week follow-up of detraining (Post-2). * = *p* < 0.05 from pre evaluation; # = *p* < 0.05 from post-1 evaluation; ¥ = *p* < 0.05 from CG; PT = patellar tendon; CG = control group; EG1 = experimental group 1; EG2 = experimental group 2.

The values recorded for the thickness and EI in PT are shown in Figure 3. Significant time x group interaction (F = 5.28; *p* = 0.001) but no inter-group (CG, EG1 and EG2) differences were found in the PT thickness. The PT thickness was 0.08 ± 0.05 cm (IC 95%, from 0.04 to 0.11 cm, *p* < 0.001, ES = 1.3) greater in EG1 in POST-1 compared to PRE and 0.06 ± 0.07 cm (IC 95%, from 0.01 to 0.11 cm, *p* = 0.011, ES = 0.9) lower in POST-2 compared to POST-1. Significant time x group interaction (F = 3.84; *p* = 0.008) were found in the EI. The EI values of EG1 were greater (*p* < 0.05) in POST-2 compared to both PRE and POST-1. Moreover, EG1 showed greater values (*p* < 0.05) than EG2 and CG in POST-2.

### 3.2. Vastus Lateralis Structure

The values obtained for the pennation angle, thickness, and fascicle length in VL are shown in Table 2. No significant differences were found among groups (CG, EG1, and EG2) in any of the assessments (PRE, POST-1, and POST-2) for any of the variables studied except in the pennation angle and the thickness of the VL distal region in POST-1 between EG1 and CG. Differences were observed intra-groups. The pennation angle of the VL distal region in EG1 was 2.25 ± 2.19° (IC 95%, from 0.4 to 4.1°, *p* = 0.021, ES = 0.9) greater in POST-1 compared to PRE, and 3.0° ± 2.19° (IC 95%, from 0.6 to 5.5°, *p* = 0.018, ES = 1.3) lower in POST-2 compared to POST-1. In addition, the thickness in the distal region was 0.22 ± 0.07 cm (IC 95%, from 0.03 to 0.41 cm, *p* = 0.026, ES = 0.9) greater in EG2 in POST-1 compared to PRE. In the same way, EG2 showed an increase of 0.22 ± 0.18 cm (IC 95%, from 0.01 to 0.44 cm, *p* = 0.042, ES = 0.6) in the thickness at 50% after the training and a decrease of 0.17 ± 0.21 cm (IC 95%, from 0.04 to 0.29 cm, *p* = 0.013, ES = 0.5) after the 6 weeks of follow-up without training.

No significant differences were found in the pennation angle at 50% and in the fascicle length at 50% and in the distal region between PRE, POST-1, and POST-2 in any of the groups. No significant differences were found in CG in any of the variables analyzed in the VL between PRE, POST-1, and POST-2.

### 3.3. Composition of the Thigh

The values recorded for the lean mass and the percentage of fat mass of the thigh are shown in Table 3. No significant differences were found in inter-group comparisons (CG, EG1, and EG2) for any of the variables studied. No significant differences were found in CG in any of the variables analyzed in the composition of the thigh between PRE, POST-1, and POST-2. Significant increases (*p* < 0.05) were found after training in lean mass in both experimental groups (EG1: diff = 0.30 ± 0.38 kg; CI 95%: from 85.6 to 520.1 kg, *p* = 0.004, ES = 0.3 and EG2: diff = 0.36 ± 0.27 kg; CI 95%: from 155.4 to 571.4 kg, *p* = 0.001, ES = 0.5). The fat percentage in EG1 and EG2 was lower in POST-1 compared to PRE (EG1: diff = 0.85 ± 0.95%; CI 95%: from 0.1 to 1.6%, *p* = 0.016, ES = 0.1 and EG2: diff = 1.02 ± 1.00%; CI 95%: from 0.3 to 1.7%, *p* = 0.002, ES = 0.2) and for both groups was greater (EG1: *p* = 0.019 and EG2: *p* = 0.015) in POST-2 compared to POST-1.

## 4. Discussion

The aim of this study was to determine the effect of the SLDSe with different technical execution times (3 s and 6 s) on the morphological and structural properties of the VL and PT and on the composition of the thigh between EG1 (executing the exercise in a time of 6 s), EG2 (executing the exercise in a time of 3 s), and CG without intervention. The results demonstrated greater values of thickness in PT, pennation angle, thickness, and fascicle length in the distal region of the VL and lean mass on the thigh in EG1 after the 6-week intervention program. EG1 and EG2 showed lower values of fat mass on the thigh after the intervention program. These results suggest that 6 weeks with 3 sessions per week with 3 series of 8 repetitions at 80% of 1-RM of the SLDSe cause an increase in the thickness of the main muscles and tendons of the knee extensor apparatus (VL and PT), considering that after 6 weeks without training, these adaptations tended to return to the initial values measured before the intervention.

Although the tendon is considered an avascular structure, it has been shown to respond to external mechanical loads by altering its biomechanical properties (Young’s modulus) and/or morphological characteristics (thickness and CSA) [27,32]. Present data showed a PT hypertrophic response associated with SLDSe performed in 6s evidenced by the greater tendon thickness with an increase of 21.8 ± 15.0% at the end of the 6 weeks of the intervention program. These data coincide with previous investigations that have managed to hypertrophy the PT, although they used at least 12 weeks of intervention programs through eccentric exercise [33,34]. Our results have important clinical relevance because we demonstrate that 6 weeks of SLDSe training performed more slowly (6 s) are enough to increase the thickness of the PT. This circumstance may be due to the fact that the time in which the tendon is subjected to external overload is greater in the repetitions performed more slowly and stimulates the synthesis process of collagen more, causing an increase in the thickness of the tendon [35]. Eccentric exercise has been one of the most widely used conservative treatment modalities for the recovery of tendinopathies in general [36] and PT tendinopathy in particular [34]. The SLDSe performed in our study (carried out on a 25° incline) offered more favorable results than the single-leg squat performed on a flat surface [36] because the load to which the PT is subjected is greater when the SLDSe is performed on a 25° incline than on a flat surface [37].

Sonoelastography has proven to be a reliable and reproducible technique in the exploration of the stiffness index of healthy PT [38]. The data show that after the 6 weeks without training, the EI increased 91.2 ± 71.4% in EG1 with respect to baseline, which seems to indicate that eccentric exercise causes different long-term adaptations in the stiffness of the PT to isometric exercise, since Kubo et al. [39] found a reduction in the stiffness of the tendon two months after completing a training program using isometric contractions. A higher value in the EI of the PT, such as that found in our study after six weeks without training, indicates greater stiffness, which may benefit the rate of force development [40], and it has been associated with better performance in agility tests, pace changes, sports with continuous stretch–shortening cycles and speed/sprint tests [41]. However, at the same time, the risk of muscle injury in the tendons with these characteristics is higher because the high stiffness makes the tendon absorb less energy and increases the forces that are generated in muscle [42].

The VL is a muscle that has a great capacity for structural adaptation to eccentric strength at the distal level [43]. Other studies have shown adaptations of the architecture of the extensor muscles of the knee after four weeks of training with eccentric exercises [44]. The results in this study have shown that the intervention program increased the thickness of the VL in EG2 (group that trained the SLDSe during 3 s) by 15.7 ± 5.9% at the distal level, and by 12.4 ± 11.7% measured at 50% of the thigh length. In addition, we also found a trend to increase VL thickness at the distal level (*p* = 0.097) and 50% of the thigh length (*p* = 0.059) in EG1 in this group to an increase of 13.4 ± 13.2% in the distal pennation angle and a tendency to increase in EG2 (*p* = 0.062). These results indicate that the mechanical stimuli induced by eccentric exercise of high intensity (80% of the 1-RM) and performed with a technical execution time of 3s may be a fundamental mechanism for VL hypertrophy. Therefore, the speed of execution of the eccentric repetition is a determining factor to achieve adaptations in the mentioned variables of the VL [45]. The results of this study have shown that regardless of the technical execution time, after six weeks of detraining, the adaptations were lost both at the tendon and muscle levels. These results are in line with other studies that claim that the muscular strength returns to control levels over several weeks of detraining [15,16].

This study showed that regardless of technical execution time of the SLDSe at the end of the intervention program, both experimental groups increased lean thigh mass (EG1 ≈4.2% and EG2 ≈5.0%). These results are in line with other research that has stated that eccentric exercise is the most effective training mode for promoting muscle growth [46]. In addition, eccentric exercise causes greater gains in muscle mass than concentric exercise because it produces a series of histochemical and metabolic substrates that induce hypertrophy [47]. Moreover, the fat mass in the thigh decreased in both experimental groups at the end of the 6-week intervention program, which indicates the importance of strength training to improve the lipid profile [48]. It was also shown that after six weeks without training, the muscle mass and fat mass values of the thigh tended to return to their initial levels.

There were some limitations to this study that deserve attention. Our data are best extrapolated to young, healthy, and physical active males. Although this population is arguably the most likely to use eccentric training, other populations such as older individuals, females, and rehabilitation patients may or may not respond in the same manner as our study cohort. We used the eccentric single-leg declined squat exercise, and some individuals may perceive this as a less practical exercise and may not extrapolate well to other sports-related activities. The eccentric training and vastus lateralis and patellar tendon adaptations only were performed on the dominant leg of the participants; the reported results may not be similar in the non-dominant leg. Finally, despite the fact that subjects have been instructed not to perform strength training outside the study protocol, their daily physical activity may affect the study results.

## 5. Conclusions

In conclusion our results indicate that the eccentric exercise of the SLDSe performed at high intensities (80% of the 1-RM) and carried out with the execution time of 6 s caused an increase of ≈21.8% in the thickness of the PT and carried out with the execution time of 3 s caused an increase of ≈15.7% in the thickness of the VL. The slower eccentric exercise of SLDSe had greater effects on the structural and elastic properties of the PT and, conversely, when it was performed more quickly, it caused greater morphological and structural adaptations in the VL musculature. In addition, it has been shown that regardless of the technical execution time, after six weeks without training, adaptations were lost both at the tendon and muscle levels. These findings suggest that SLDSe is effective for both PT and VL hypertrophy as well as helping to lose fat mass in the thigh.

As practical application, the knowledge of the effects of an eccentric training program lasting 6 weeks with different execution times on the morphological and structural properties of VL and PT, which are two of the main protagonists of the knee extensor apparatus, is essential both for the treatment modalities for recovery from injuries and for improving the performance of athletes. Current results suggest that 6 weeks of eccentric training on the dominant leg of the SLDSe exercise produces increases in VL and PT thickness and thigh muscle mass, which could be used when the goal is to improve muscle strength at the performance level or to produce muscle and tendon adaptations in recovery from injuries.

## Figures and Tables

**Table 1:** Descriptive characteristics of the subjects.

	CG (*n* = 13)	EG1 (*n* = 11)	EG2 (*n* = 12)
Age (years)	20.77 ± 1.88	21.55 ± 2.46	21.08 ± 1.24
Weight (kg)	69.84 ± 10.89	71.85 ± 11.82	71.27 ± 8.28
Height (cm)	1.75 ± 0.06	1.76 ± 0.07	1.74 ± 0.07
Fat percentage (%)	18.99 ± 7.02	18.91 ± 05.04	19.54 ± 4.81

Presented as mean ± SD. CG = control group; EG1 = experimental group 1; EG2 = experimental group 2.

**Table 2:** Morphological properties of VL in response to six weeks of eccentric single-leg decline squat exercise training (Post-1) two technical execution times (EG1 = 6 s and EG2 = 3 s) and 6-week follow-up of detraining (Post-2).

	PRE	POST-1	POST-2	*p*-Value	Timeline Effect		Time × Group Interaction	
					F	*p*-Value	F	*p*-Value
Pennation angle VL distal (°)								
CG	15.92 ± 2.69	16.15 ± 3.29	17.15 ± 2.19	0.437	3.94	0.032	3.83	0.034
EG1	17.38 ± 2.39	19.63 ± 2.88 *¥	16.63± 1.77 #	0.025				
EG2	15.50 ± 3.24	17.10 ± 3.96	17.40 ± 2.98	0.108				
Thickness VL distal (cm)								
CG	1.53 ± 0.32	1.39 ± 0.33	1.48 ± 0.27	0.267	1.26	0.299	5.01	0.014
EG1	1.61 ± 0.45	1.79 ± 0.41 ¥	1.68 ± 0.40	0.244				
EG2	1.42 ± 0.24	1.63 ± 0.26 *	1.55 ± 0.36	0.088				
Fascicle length VL distal (cm)								
CG	5.65 ± 1.14	5.45 ± 0.99	5.30 ± 0.63	0.716	0.47	0.628	0.06	0.993
EG1	5.15 ± 2.47	5.10 ± 0.56	4.89 ± 0.85	0.860				
EG2	5.51 ± 1.19	5.57 ± 1.35	5.39 ± 0.90	0.877				
Pennation angle VL 50% (°)								
CG	15.46 ± 3.89	15.62 ± 3.69	15.46 ± 3.13	0.954	2.53	0.099	0.60	0.662
EG1	14.75 ± 2.76	16.38 ± 2.62	16.63 ± 2.07	0.168				
EG2	14.20 ± 2.97	15.40 ± 2.46	15.10 ± 2.51	0.297				
Thickness VL 50% (cm)								
CG	2.09 ± 0.39	2.08 ± 0.33	2.07 ± 0.33	0.961	4.34	0.023	2.72	0.083
EG1	1.90 ± 0.38	2.13 ± 0.36	2.08 ± 1.82	0.164				
EG2	2.02 ± 0.71	2.25 ± 0.30 *	2.08 ± 0.32 #	0.016				
Fascicle length VL 50% (cm)								
CG	8.05 ± 1.74	7.99 ± 1.72	7.98 ± 1.82	0.997	0.78	0.470	0.35	0.843
EG1	7.04 ± 3.56	8.01 ± 1.85	7.62 ± 1.01	0.353				
EG2	7.84 ± 03.40	8.33 ± 1.79	7.92 ± 1.26	0.455				

* = *p* < 0.05 from pre-evaluation; # = *p* < 0.05 from post-1 evaluation; ¥ = *p* < 0.05 from CG; VL = vastus lateralis; CG = control group; EG1 = experimental group 1; EG2 = experimental group 2.

**Table 3:** Composition of the thigh in response to six weeks of eccentric single-leg decline squat exercise training (Post-1) with two technical execution times (EG1 = 6 s and EG2 = 3 s) and 6-week follow-up of detraining (Post-2).

	PRE	POST-1	POST-2	*p*-Value	Timeline Effect		Time × Group Interaction	
					F	*p*-Value	F	*p*-Value
Lean mass thigh (kg)								
CG	7.01 ± 1.16	7.03 ± 1.17	7.16 ± 1.21	0.164	10.75	*p* < 0.001	2.89	0.029
EG1	7.43 ± 1.19	7.73 ± 1.21 *	7.51 ± 1.20 #	0.005				
EG2	7.19 ± 0.77	7.55 ± 0.88 *	7.36 ± 0.74	0.001				
Fat percentage thigh (%)								
CG	20.36 ± 8.44	20.36 ± 8.06	20.57 ± 7.95	0.822	17.90	*p* < 0.001	2.88	0.030
EG1	19.30 ± 5.80	18.45 ± 6.21 *	19.42± 6.10 #	*p* < 0.001				
EG2	19.89 ± 5.55	18.88 ± 4.78 *	19.83 ± 4.80 #	*p* < 0.001				

* = *p* < 0.05 from pre evaluation; # = *p* < 0.05 from post-1 evaluation; CG = control group; EG1 = experimental group 1; EG2 = experimental group 2.
